# Education of doctors providing service to LGBTQ patients

**Published:** 2015-12-11

**Authors:** Adem Parlak, Sedat Develi, Nehir Parlak, Yusuf Emrah Eyi

**Affiliations:** 1 Department of Medical Education, Hacettepe University Faculty of Medicine, Ankara, Turkey; 2 Department of Anatomy, Gulhane School of Medicine, Ankara, Turkey; 3 Department of Dermatology, Etimesgut Public Hospital, Ankara, Turkey; 4 Department of Emergency Medicine, President Guard Regimen, Ankara, Turkey

We read Beagan et al.’s article describing the challenges faced by LGBTQ patients during examinations. The problems which LGBTQ patients encounter because of the way that doctors respond to their situation were discussed in detail. It was also mentioned that during their college education physicians providing health care to these patients were not informed enough about the diseases of this population.^1^

We want to share our comments on Beagan et al.’s study. We believe that designing a study with only the information obtained from the medical examiner is a limitation because prejudice has been understood from the doctors’ perspective. We feel strongly that the problems experienced by both groups should be described in more detail.

Also, Beagan et al. have mentioned that problems faced by this patient group have been better addressed in the new medical school training programs. But the current study was conducted with physicians trained with previous medical education programs. Current physicians need in-service training programs related to the care of LGBTQ patients. In addition, routine and comprehensive support for these patients with social workers, psychologists and sexual health professionals is crucial for both the individuals and the community as a whole.^2^,^3^

We thank Beagan et al. for the contributions they have made and for the opportunity to respond to their article.
